# Exposure to silicates and systemic autoimmune-related outcomes in rodents: a systematic review

**DOI:** 10.1186/s12989-021-00439-6

**Published:** 2022-01-07

**Authors:** Lisa M. F. Janssen, Manosij Ghosh, Frauke Lemaire, K. Michael Pollard, Peter H. M. Hoet

**Affiliations:** 1 Laboratory of Toxicology, Unit of Environment and Health, Department of Public Health and Primary Care, KU Leuven, Leuven, Belgium; 2 Laboratory of Respiratory Diseases and Thoracic Surgery (BREATHE), KU Leuven, Leuven, Belgium; 3 Department of Molecular Medicine, Scripps Research, La Jolla, CA 92037 USA

**Keywords:** Environmental exposure, Occupational exposure, Silica, Asbestos, Autoimmunity, Systemic autoimmunea diseases, Immune-mediated diseases, Rodents, Mice, Rat

## Abstract

**Background:**

Autoimmunity can result from the interplay between genetic background and effects of environmental and/or occupational exposure to hazardous materials. Several compounds, including silica dust, have been linked with systemic autoimmunity and systemic autoimmune diseases, based on epidemiological evidence. For asbestos, a strong link with systemic autoimmune diseases does not yet exist, however, several studies have documented features of autoimmunity following asbestos exposure. Even so, human studies are limited in their ability to identify and examine isolated exposures, making it difficult to demonstrate causation or to assess pathogenic mechanisms. Therefore, this systematic review examines the existing animal evidence regarding autoimmunity and exposure to silicates (silica and asbestos).

**Methods:**

PubMed and EMBASE were systematically searched for peer-reviewed studies examining systemic autoimmune disease-related outcomes after silicate exposure in rodents. Literature databases were searched up to September 2021 for studies written in English and where the full text was available. Search strings were established based on a PECO (Population, Exposure, Comparator, Outcome) format. After title, abstract, and full-text screening, thirty-four studies were identified for further analysis. Quality assessment through ToxR tool and qualitative analysis of the results was performed.

**Results:**

Although there was significant heterogeneity in the included studies in terms of exposure protocol and genetic background of the rodent models used, it was noted that both genetic background and exposure to silicates [(crystalline) silica and asbestos] are highly relevant to the development of (sub-) clinical systemic autoimmune disease.

**Conclusion:**

Parallels were observed between the findings from the animal (this review) and human (epidemiological) studies, arguing that experimental animal models are valuable tools for examining exacerbation or development of autoimmune disease after silicate exposure. However, genetic background and synergism between exposures should be considered in future studies.

**Supplementary Information:**

The online version contains supplementary material available at 10.1186/s12989-021-00439-6.

## Introduction

Autoimmune diseases (ADs) are a poorly understood group of chronic diseases, resulting from a self-reactive immune response [1]. Being chronic and incurable, ADs constitute a major public health problem with considerable human suffering and societal costs. Based on the extent of tissue involvement, ADs are categorized into organ-specific [e.g. type I diabetes (T1D)] and systemic [e.g. systemic lupus erythematosus (SLE), rheumatoid arthritis (RA), systemic sclerosis (SSc)] autoimmune diseases.

Autoimmune diseases are often described as idiopathic or of unknown cause [2]. However, some autoimmune responses seem to emerge from the interaction between environmental triggers together with disease susceptible genotypes, but little is known about the cellular or molecular basis of this interaction. Specifically, environmental or occupational particulate exposures, such as silica dust, asbestos and cigarette smoke, have been linked with systemic autoimmune diseases (SAIDs) in human cohorts or epidemiological studies [3].

Over the years, treatment options for SAIDs have moved from the use of systemic glucocorticoids to biological agents, which provide a more targeted approach to the disease [4]. However, effective therapy development remains a significant challenge, due to disease heterogeneity and variable disease mechanisms [5]. While this highlights the urgent need for more effective treatments, their development will require a better understanding of underlying disease mechanisms. Although some of the general principles of disease pathogenesis, like the recognition of self or foreign molecules by innate sensors, have been elucidated [6], the initiating steps that drive auto-reactivity remain poorly understood.

The Earth’s crust is made up of almost 60% of crystalline silica, and it serves as a key industrial product [7]. A large group of people working in different industries such as sandblasting, sand extraction [8] and construction, are exposed to silica-containing dusts. Asbestos, on the other hand, is a generic term that represents different mineral silicates that can be grouped into two distinct classes based on their shape, physical, and chemical properties, i.e. serpentine asbestos, including chrysotile asbestos; and amphibole asbestos, including amosite, crocidolite, tremolite, actinolite, and anthophyllite asbestos [9]. Different types of asbestos can be confidently linked with, among others pathologies, mesothelioma and asbestosis [10, 11]. Extensive research has also revealed adverse health effects related to serpentine/chrysotile asbestos exposure [12, 13]. In 1977, the International Agency for Research on Cancer (IARC) classified asbestos as a human carcinogen [14]. Although its use is strongly regulated in several countries, it is still not banned in about 70% of the world [15]. In addition, a new asbestos-related health hazard is arising from exposure to the other form of asbestos, the amphibole group, which are often referred to as naturally occurring asbestos fibers. Environmental exposure to asbestos-containing dust, as a result of climate change, is increasing due to increasing dryness in regions where asbestos is present in the bedrock [16].

Despite a body of evidence supporting an association between asbestos exposure and systemic autoimmunity, including the presence of antinuclear antibodies (ANA) [17–20], a strong epidemiological link with specific SAIDs has yet to be made. This contrasts with silica, where there is considerable evidence linking exposure to several SAIDs. This was highlighted in a recent review of epidemiologic evidence for environmental factors in human autoimmune diseases [3]. It was concluded that exposure to crystalline silica contributes to the development of several systemic autoimmune diseases, including SLE, RA, SSc, and antineutrophil cytoplasmic antibody (ANCA)-related vasculitis. Research has shown that silicate dusts increase the production of autoantibodies, possibly through the production of excess cellular debris in the context of a highly inflammatory environment [21–23]. In addition, recent reviews [24, 25] were able to identify a series of molecular and cellular events that are common to several particles, including cigarette smoke, crystalline silica, and asbestos, linked to systemic autoimmunity. However, evidence identifying specific mechanisms for specific exposure compounds that drive systemic autoimmunity is still lacking, and it is not known whether there is a common response to inhaled mineral dusts.

Linking particulate exposure with human systemic autoimmune disease manifestations is difficult because of the inherent limitations of epidemiological studies to draw causal conclusions. Additionally, human populations are rarely exposed to a single xenobiotic compound, and exposure and disease onset are not often clearly related, making it difficult to identify the compounds of significance. In addition, the specific mechanisms leading to SAIDs and the effects of environmental and occupational exposures on those mechanisms remain largely unknown. However, many of these limitations can be overcome by studying experimental animal models to understand the interaction of the environment with the specific players in the story of systemic autoimmunity.

In general, there appears to be little consensus on the parameters necessary to establish an experimental model of silica- or asbestos-induced systemic autoimmunity. This stands in contrast to induction of silicosis in animal models where there is vast array of literature on both in vitro and in vivo approaches; although this does not mean there are consensus protocols. Moreover, it is still unknown whether the diagnosis of silicosis is essential for development of autoimmunity, as suggested in some studies [26–28]. Therefore, the objectives of the current systematic review was to test the hypothesis that current data from experimental animal models establish some of the parameters for induction of silicate-induced autoimmunity but provide no consensus and tell us little of the cellular and molecular mechanisms that lead to disease.

## Methods

The methodology of this systematic review was adopted from the Systematic Review Protocol for Animal Intervention Studies [29], which is a previously published protocol analogous to the Cochrane review protocol. It is described in detail in the sections below.

### Literature searches and screening

#### Search strategy

A PECO (Population, Exposure, Comparator, Outcome) format was developed to frame the research question and guide the screening of relevant studies (Table 1). To determine the outcomes that define “systemic autoimmunity”, a selection was made of the most relevant SAIDs and their features. To do so, different review papers were consulted [3, 30].

**Table 1:** The PECO (population, exposure, comparator, outcome)

Variable	Description
Population	Experimental rodents
Exposure	Any administered dose of the selected compounds (silica dust (amorphous or crystalline) or asbestos)) as singular compounds
Comparator	Exposure to vehicle-only or untreated control
Outcome	Systemic autoimmune diseases: systemic sclerosis, systemic lupus erythematosus, rheumatoid arthritis, Sjögren’s syndrome, antineutrophil cytoplasmic antibody (ANCA)-associated vasculitis, autoimmune myositis Systemic autoimmunity features: kidney pathology/glomerulonephritis, lung pathology, autoantibodies, changes in serum immunoglobulins, changes in serum cytokines, proteinuria, skin involvement, joint involvement

Based on this PECO, search strings to conduct a systematic literature search were designed to capture all potentially pertinent studies. The literature search was conducted using two online scientific databases, MEDLINE/PubMed and Embase, for studies published before September 17, 2021. Limits were set for language (only English studies) and publication type (No reviews). We did not set limits for publication year at this point. Three groups of keywords were used; (a) the names of the selected particulate compounds, (b) keywords concerning systemic autoimmunity and (c) keywords to search for animal exposure studies. To detect all rodent studies, animal search filters [31, 32] were modified to only include rodents. All terms were searched using both controlled vocabulary [Medical Subject Headings (MeSH) in PubMed and Emtree terms in Embase], if MeSH or Emtree term was available, and free text words in titles and abstracts. The exposure compounds were selected based on a review from an expert panel workshop by the National Institutes of Environmental Health Sciences (NIEHS) [3]. The search strings were approved by a librarian and are provided in the additional files (see Additional file 2: Table 1). Searches from both databases were transferred to the reference manager EndNote, and duplicates were removed.

#### Study selection

The study selection consisted of two screening phases. The first selection was based on title and abstract screening, and the second selection was based on a full-text screening.

##### Title and abstract screening

Title and abstract were screened based on the following pre-selected inclusion criteria: (a) in vivo animal intervention study using rodents and (b) exposure to (one or more of) the selected occupational particulate compounds. Specifics on the forms and types of silica and asbestos that were to be included or excluded are described in more detail in the additional files (see Additional file 1). Based on the title and abstract, the studies were categorized as "yes" (strong evidence in support of inclusion), "maybe" (evidence inadequate for clear selection/rejection), or "no" (strong evidence against inclusion). Studies without abstract available were automatically categorized as "no".

Title and abstract screening were performed by two independent reviewers (L.J. and F.L.). using the online software tool Rayyan. Disagreements were discussed between the two reviewers to reach agreement. Remaining discrepancies were resolved by consulting a third reviewer (M.G.). Percentage of agreement and Cohen's kappa were calculated to give an idea of the agreement between the selection of the reviewers.

##### Full-text screening

At the full-text level, studies were checked for the aforementioned criteria, and for two extra criteria: (a) systemic autoimmune outcome and (b) publication type (only journal articles that present original unique data). Since the number of studies was rather limited, and we noticed a plethora of different approaches to assess “systemic autoimmunity”, we did not set very strict criteria for this parameter. Selected systemic autoimmune parameters are based on the PECO table, and are described in more detail in the inclusion- and exclusion criteria in the additional files (see Additional file 1). In brief, there were two different options for inclusion for this point; either the paper discussed clinical and pre-clinical signs of systemic autoimmunity, or the paper examined mechanisms of silicate-induced systemic autoimmunity. In addition, it was decided to put a date limit at this point, including only manuscripts from 2000 or later. Disagreements were discussed by the two reviewers to reach agreement (L.J. and F.L.); if consensus could not be reached, the third reviewer (M.G.) resolved the differences.

#### Study characteristics and data extraction

After the dataset of relevant studies was established by performing the previously described steps, several study characteristics and data items of each study were extracted and summarized in a data extraction table. The following characteristics were extracted from the included studies: bibliographic data (authors, title, and publication year), animal model characteristics (species, strain, sex, age), exposure compound, exposure route, dosages, frequency of exposure, time between dosing and sacrificing animals, reported outcome measures, and results of systemic autoimmune endpoints. Simplified data tables are shown in the results section.

To assess the quality of the included animal studies, the Toxicological data Reliability Assessment Tool (ToxRTool) for in vivo studies list was used [33], which provides more detail compared to the SYRCLE's risk of bias tool [34]. Based on the score from 21 criteria (see Additional file 3: Table 2), studies were assigned to one of the four Klimisch score levels [35], as follows: 1 (reliable without restrictions), 2 (reliable with restrictions), 3 (not reliable) and 4 (not assignable). Quality assessment was performed by two independent reviewers (L.J. and F.L.), and in case of doubt, agreement was reached by mutual discussion. Several questions caused some confusion among the reviewers, this was resolved by agreeing on some additional criteria specifically applicable to the data used in this review. To assess question 2 regarding the purity of the substance, an agreement was made to check if the paper mentions endotoxin testing of the substance. If they mention nothing about the purity of the sample, a 0 was given. If they mention that the substance was washed and/or baked to purify it but there was no testing of the presence of endotoxin afterwards, a 0 was given. If they mention purifying the substance and testing for endotoxin presence afterwards, a 1 was given. For question 4 regarding the physico-chemical characteristics of the substance, the reviewers agreed to give 1 point when a standardized form of the test substance was used that has already been characterized. If a non-standard form was used and information about the physico-chemical characteristics was given in the paper, 1 point was given. To assess question 16 the reviewers agreed to give a point if only one dose was administered or if the paper specifically mentions that they made a fresh suspension of the test substance. Lastly, for question 20, the reviewers agreed to give 1 point if the papers mentions randomization or blinding when describing their experimental design. If they mention neither, 0 points were given.

**Table 2:** Bibliographical information of included studies

	First and last author	Year	Title	PubMed ID	References
Asbestos	Blake, D. J. and Pfau, J. C	2008	Autoantibodies from mice exposed to Libby amphibole asbestos bind SSA/Ro52-enriched apoptotic blebs of murine macrophages	18295955	[36]
Asbestos	Pfau, J. C. and Blake, D. J	2008	Asbestos-induced autoimmunity in C57Bl/6 mice	18569382	[37]
Asbestos	Pfau, J. C. and Sentissi, J. J	2011	Alteration of fibroblast phenotype by asbestos-induced autoantibodies	21457077	[38]
Asbestos	Salazar, K. D. and Luebke, R. W	2012	Effects of Libby amphibole asbestos exposure on two models of arthritis in the Lewis rat	22480172	[39]
Asbestos	Salazar, K. D. and Luebke, R. W	2013	Evaluation of anti-nuclear antibodies and kidney pathology in Lewis rats following exposure to Libby amphibole asbestos	23256773	[40]
Asbestos	Ferro, A. and Pfau, J. C	2014	Amphibole, but not chrysotile, asbestos induces anti-nuclear autoantibodies and IL-17 in C57BL/6 mice	24164284	[41]
Asbestos	Pfau, J. C. and Marcum, R	2014	Activation and trafficking of peritoneal B1a B-cells in response to amphibole asbestos	23746315	[42]
Asbestos	Zebedeo, C. N. and Pfau, J. C	2014	Erionite induces production of autoantibodies and IL-17 in C57BL/6 mice	24518925	[43]
Asbestos	Gilmer, J. and Pfau, J. C	2016	Libby amphibole-induced mesothelial cell autoantibodies promote collagen deposition in mice	27106292	[44]
Asbestos	Pfau, J. C. and Keil, D. E	2017	Comparative health effects in mice of Libby amphibole asbestos and a fibrous amphibole from Arizona	28870655	[45]
Asbestos	Christofidou-Solomidou, M. and Pfau, J. C	2019	Synthetic secoisolariciresinol diglucoside (LGM2605)inhibits Libby amphibole fiber-induced acute inflammation in mice	31022494	[46]
Silica	Brown, J. M. and Holian, A	2003	Silica accelerated systemic autoimmune disease in lupus-prone New Zealand mixed mice	12605693	[47]
Silica	Ezendam, J. and Pieters, R	2003	Immunomodulatory effects of tetrachlorobenzoquinone, a reactive metabolite of hexachlorobenzene	12807351	[48]
Silica	Brown, J. M. and Holian, A	2004	Immunoglobulin and Lymphocyte Responses Following Silica Exposure in New Zealand Mixed Mice	15204774	[49]
Silica	Pfau, J. C. and Holian, A	2004	Silica-exposed mice generate autoantibodies to apoptotic cells	14751672	[21]
Silica	Brown, J. M. and Holian, A	2005	Effects of rottlerin on silica-exacerbated systemic autoimmune disease in New Zealand mixed mice	16040631	[50]
Silica	Al-Mogairen, S. M. and Gad El Rab, M. O	2009	Induction of autoimmunity in Brown Norway rats by oral and parenteral administration of sodium silicate	19318393	[51]
Silica	Al-Mogairen, S. M	2011	Role of sodium silicate in induction of scleroderma-related autoantibodies in brown Norway rats through oral and subcutaneous administration	20049452	[52]
Silica	Wilfong, E. R. and Chapman, G. D	2011	The acute and long-term effects of middle east sand particles on the rat airway following a single intratracheal instillation	21899408	[53]
Silica	Chen, Y. and Chen, J	2013	Neutralization of interleukin-17A delays progression of silica-induced lung inflammation and fibrosis in C57BL/6 mice	24291675	[54]
Silica	Bates, M. A. and Pestka, J. J	2015	Silica triggers inflammation and ectopic lymphoid neogenesis in the lungs in parallel with accelerated onset of systemic autoimmunity and glomerulonephritis in the lupus-prone NZBWF1 mouse	25978333	[55]
Silica	Bates, M. A. and Pestka, J. J	2016	Silica-triggered autoimmunity in lupus-prone mice blocked by docosahexaenoic acid consumption	27513935	[56]
Silica	Engelmann, R. and Müller-Hilke, B	2017	Experimental silicosis does not aggravate collagen-induced arthritis in mice	28285600	[57]
Silica	Bates, M. A. and Pestka, J. J	2018	Dietary docosahexaenoic acid prevents silica-induced development of pulmonary ectopic germinal centers and glomerulonephritis in the lupus-prone NZBWF1 mouse	30258439	[58]
Silica	Mayeux, J. M. and Pollard, K. M	2018	Silicosis and Silica-induced autoimmunity in the diversity outbred mouse	29755467	[59]
Silica	Bates, M. A. and Pestka, J. J	2019	Mapping of dynamic transcriptome changes associated with silica-triggered autoimmune pathogenesis in the lupus-prone NZBWF1 mouse	30984195	[60]
Silica	Benninghoff, A. D. and Pestka, J. J	2019	Docosahexaenoic Acid Consumption Impedes Early Interferon- and Chemokine-Related Gene Expression While Suppressing Silica-Triggered Flaring of Murine Lupus	31921124	[61]
Silica	Foster, M. H. and Clark, A. G	2019	Silica Exposure Differentially Modulates Autoimmunity in Lupus Strains and Autoantibody Transgenic Mice	31632407	[62]
Silica	Lescoat, A. and Lecureur, V	2020	Crystalline Silica Impairs Efferocytosis Abilities of Human and Mouse Macrophages: Implication for Silica-Associated Systemic Sclerosis	32133004	[63]
Silica	Rajasinghe, L. D. and Pestka, J. J	2020	Omega-3 fatty acid intake suppresses induction of diverse autoantibody repertoire by crystalline silica in lupus-prone mice	32903098	[64]
Silica	Chauhan, P. S. and Pestka, J. J	2021	Rapid Induction of Pulmonary Inflammation, Autoimmune Gene Expression, and Ectopic Lymphoid Neogenesis Following Acute Silica Exposure in Lupus-Prone Mice	33732257	[65]
Silica	Pestka, J. J. and Harkema, J. R	2021	Omega-3 Polyunsaturated Fatty Acid Intervention Against Established Autoimmunity in a Murine Model of Toxicant-Triggered Lupus	33897700	[66]

An overview of the data extracted of the included studies can be found in the Additional file 4: Table 3

## Results

### Included studies and their characteristics

**Fig. 1:**
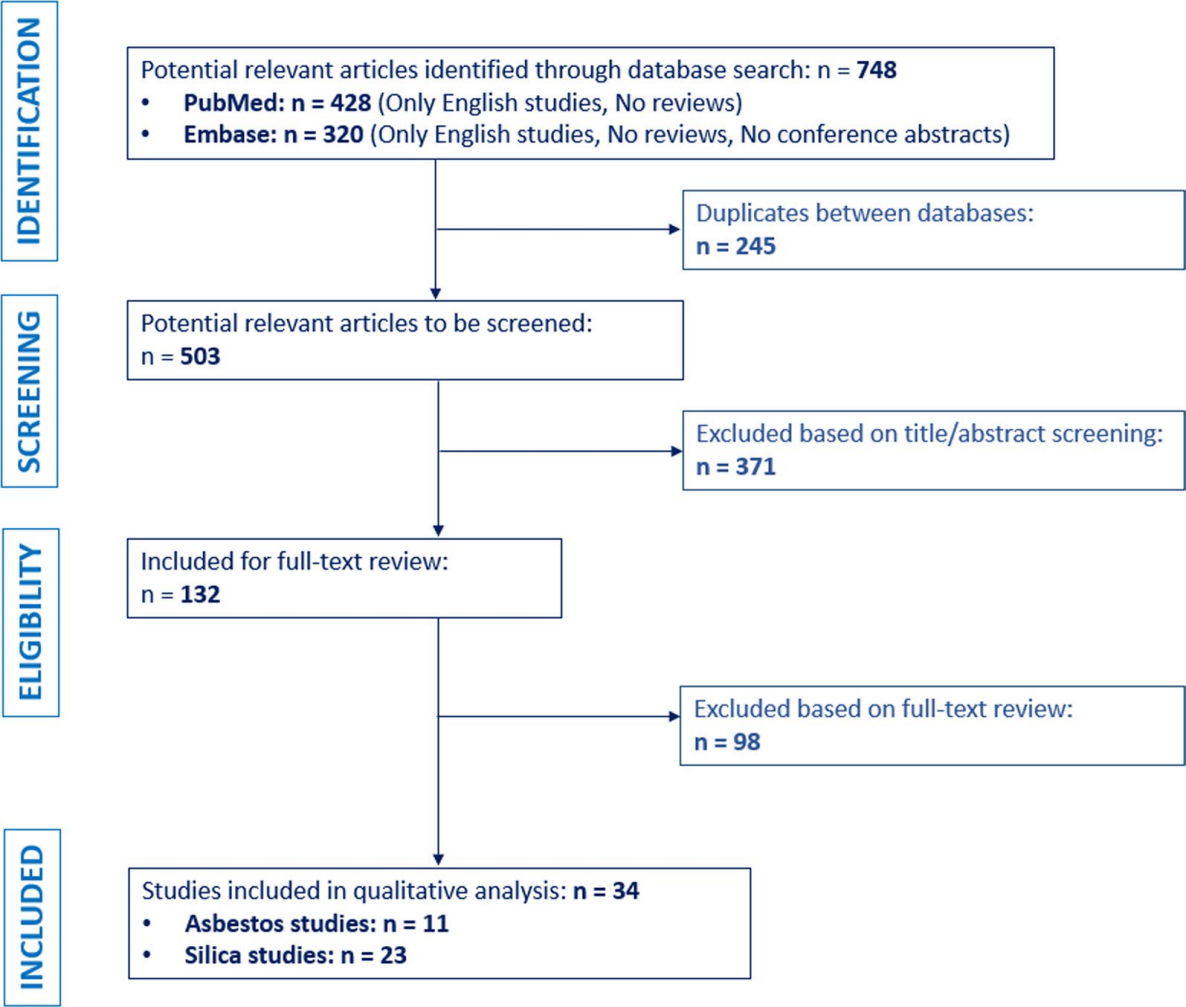
Process of inclusion and exclusion of studies

As shown in Fig. 1, the literature search yielded a total of 503 studies after duplicates were removed. After the title- and abstract screening, 132 studies were moved forward for full-text screening. From this dataset of studies, 98 studies were removed based on full-text screening, resulting in a total of 34 studies to be included in the qualitative analysis for this review. Data was extracted from the included studies on silica (n = 23) and asbestos (n = 11). Percentage of agreement of the title/abstract screening was calculated to be 84%. In addition, the Cohen’s kappa coefficient to assess inter-rater reliability for title- and abstract screening was calculated to be 0.70.

Bibliographical information on the included studies is presented in Table 2.

Based on the ToxR tool quality assessment, most of the studies (19/34) were categorized in Klimisch level 1 (reliable without restrictions). However, 12 studies were classified in Klimisch level 3, often because the key question about the plausibility of study design and results, could not be answered positively. The main reason was a lack of reporting of blinding and randomization. The grading score of ToxRTool criteria for each study is provided in Additional file 5: Table 4.

### Animal characteristics

The characteristics of the animals used in the included studies are shown in Table 3. The most commonly studied species was mouse [N = 29 (85%)]. The only other species used in the included study, was rat [N = 5 (15%)]. In the majority of the included studies [N = 20 (59%)], only female animals were included. Only two studies exclusively used male animals, whereas 10 studies included both sexes, and 4 studies did not specify the sex of the included animals.

**Table 3:** Animal characteristics in included studies

Animal characteristics		
	Asbestos (N = 11)	Silica (N = 23)
Animal species and strain		
Mouse	**9**	**20**
C57BL/6	*9 *^*(36–38, 41, 42, 44–46, 67)*^	*5 *^*(54, 55, 62, 63, 68)*^
NZM 2410	*0*	*4 *^*(21, 47, 49, 50)*^
NZWB/F1	*0*	*9 *^*(55, 56, 58, 60, 61, 65, 66, 69)*^
BXSB	*0*	*2 *^*(62, 68)*^
Other	*0*	*5 *^*(48, 56, 57, 59, 62)*^
Rat	**2**	**3**
Lewis	*2 *^*(40, 52)*^	*0*
Brown Norway	*0*	*2 *^*(51, 52)*^
Sprague–Dawley	*0*	*1 *^*(53)*^
Other	*0*	*0*
Other	**0**	*0*
Sex		
Female	**6**	**14**
Male	**0**	**1**
Both	**3**	**7**
Not specified	**2**	**2**

Values are presented as numbers

Number of studies per species and sex are indicated in Bold. The Number of studies per strain are italics. The specific studies are included in superscript

In the silica studies, there was a significant focus on the use of autoimmune-prone animals like New Zealand Mixed (NZM) mice or BXSB mice. These animals spontaneously develop autoimmunity over time and are used to examine whether certain compounds can exacerbate, suppress, or accelerate disease onset. The asbestos studies, on the other hand, mainly used healthy C57BL/6 mice.

#### Choice of compound and exposure protocols

The type of silica or asbestos particles/fibers that were used, are presented in Table 4. The most commonly used type of asbestos was the Libby (six-mix) amphibole asbestos [N = 10 (91%)]. Only two studies included chrysotile asbestos fibers, also known as “white asbestos”, in their study. The most commonly used type of silica, was the well characterized reference material Min-U-Sil 5®. The European reference material DQ10 was used in only one of the included studies. Five of the included studies mentioned using “crystalline silica” or “cSiO_2_” in the manuscript, but did not further specify the type of particles used. None of the included studies used amorphous silica. Two of the included studies used sodium silicate.

**Table 4:** Particle/fibre types in the included studies

Particle/fibre types	
Asbestos	N = 11
Amphibole	
Amosite	2
Tremolite	4
Libby	10
Other	1
Serpentine	
Chrysotile	2
Silica	N = 23
Crystalline silica	
Min-U-Sil 5	14
DQ10	1
Not specified	5
Amorphous silica	0
Sodium silicate	2
Not specified	1

Besides the specific particles or fibers used in the studies, other parameters of the exposure procedure are relevant for the outcomes as well. Exposure routes, repeated versus single dosing and duration of experiment (time from exposure to collection of samples) are presented in Table 5. In terms of exposure route, there was a significant difference between the asbestos and silica studies, as for the application of asbestos, the main exposure route [N = 10 (83%)] was intratracheal instillation, while for the application of silica, the main exposure route [N = 14 (60%)] was intranasal instillation. In the asbestos studies, single, double and repeated dosing was used, while in the silica studies, the majority of the studies used a single instillation of silica. A few papers used 4 instillations divided over 4 weeks, with a 1-week interval.

**Table 5:** Parameters of exposure protocols used in included studies

Exposure protocol		
	Asbestos (N = 11)	Silica (N = 23)
Exposure route		
Intratracheal instillation	9	1
Intranasal instillation	0	14
Oropharyngeal/transoral instillation	1	7
Intraperitoneal injection	2	0
Subcutaneous injection	0	3
Repeated dosing or single dosing		
Single	3	14
Double	6	3
Repeated (> 2x)	2	8

### Endpoints of systemic autoimmunity in asbestos-exposed rodents

Clinical outcomes, inflammatory biomarkers and mechanistic evidence found as a result of asbestos exposure, is discussed in the following sections.

#### Lung pathology

Lung collagen was observed after exposure to different types of amphibole asbestos in C57BL/6 mice [45]. Different types of amphibole asbestos induced lung injury, represented by increased lactate dehydrogenase (LDH) activity and total protein levels in bronchoalveolar lavage fluid (BALF) in arthritis models in Lewis rats [39].

#### Kidney pathology

Impairment of kidney function, shown by induction of proteinuria, immune complex deposition, and glomerulonephritis was shown to arise in C57BL/6 mice after amphibole asbestos exposure [37, 42, 45].

#### Serum autoantibodies

Different types of amphibole asbestos were observed to induce antinuclear antibodies (ANA) including anti-SS-A/Ro and anti-dsDNA [37] as well as autoantibodies against vimentin and fibroblasts, which were associated with lung fibrosis [38], in C56BL/6 mice. Other data [41, 43] showed that even a low dose of amphibole asbestos fibers can induce ANA in exposed C57BL/6 mice. Besides amphibole asbestos, also erionite fibers were observed to induce ANA using similar exposure conditions [43]. ANA were also increased in amphibole asbestos-exposed Lewis rats [40], and in arthritis models in Lewis rats, induced by an intraperitoneal injection of bacterial cell wall peptidoglycan-polysaccharide (PG-PS) or intradermal injection of bovine nasal collagen [39]. Chrysotile asbestos, on the other hand, was not able to induce ANA in C57BL/6 mice [41, 43].

Blake et al. [36] reported that apoptotic blebs containing SS-A/Ro52 in mouse macrophages are recognized by autoantibodies from asbestos-exposed mice.

Serum antibodies from amphibole asbestos-instilled mice were shown to bind to mouse fibroblasts, which was suggestive of anti-fibroblast antibodies [38]. Furthermore, these antibodies induced expression of smooth muscle α-actin (SMA) in primary lung- and skin-fibroblasts.

In addition, mesothelial cell autoantibodies (MCAA) were observed in serum of amphibole asbestos exposed C57BL/6 mice [44, 45].

#### Systemic inflammatory biomarkers

Zebedeo et al. [43] reported an increase in serum immunoglobulin G (IgG) subtype 1 (IgG1), IgG2a, and immunoglobulin M (IgM) after erionite and Korean tremolite amphibole asbestos exposure. However, Pfau et al. [37] observed an overall decrease in serum IgG in amphibole asbestos exposed C57BL/6 mice. Also Ferro et al. [41] reported decreases in serum IgG1 titers of C56BL/6 mice after amphibole asbestos and chrysotile asbestos exposure. Cytokines typical for the Th17 response, such as interleukin-17 (IL-17), interleukin-6 (IL-6), and tumor necrosis factor-alpha (TNF-α), were triggered by amphibole asbestos exposure in C57BL/6 mice [45]. Other studies observed that amphibole and erionite, but not chrysotile, asbestos induced an increase of IL-17 in serum of C57BL/6 mice [41, 43].

#### Lymph nodes, spleen and peritoneal cavity

Amphibole asbestos-exposed C57BL/6 mice showed changes in percentages of CD25 + T-suppressor cells and B1a B-cells in lymph nodes compared to non-exposed control mice [37]. B1a B-cells (CD19 + , CD5 + , CD1d +) were increased in spleen in both chrysotile and amphibole asbestos exposed C57BL/6 mice in percentages of CD25 + T-suppressor cells and B1a B-cells in lymph nodes compared to non-exposed control mice. B1a B-cells (CD19 + , CD5 + , CD1d +) were increased in spleen in both chrysotile and amphibole asbestos C57BL/6 exposed mice [41]. Regulatory B cells (IgM + , CD5 + , CD11b + , CD1d +), were decreased in spleen of amphibole asbestos exposed C57BL/6 mice [42]. Spleen weight was reported to be increased by amphibole asbestos exposure in C57BL/6 mice [46].

In the peritoneal cavity, amphibole asbestos induced a decrease of B1a B cells in C57BL/6 mice [42, 46].

### Endpoints of systemic autoimmunity in silica dust-exposed rodents

Clinical outcomes, inflammatory biomarkers and mechanistic evidence found as a result of silica dust exposure, is discussed in the following sections.

#### Lung pathology

A single dose of crystalline silica resulted in fibrotic lesions with excess collagen deposition and increased inflammatory infiltrates in the lung of lupus-prone NZM 2410 mice [47]. In addition, crystalline silica induces lung infiltration of inflammatory cells and alveolar septal thickening only one week after exposure in C57BL/6 mice. After three weeks, collagen deposition and fibrous bands were observed in these mice as well [54]. Inflammatory cell infiltration and lung pathology was also observed in crystalline silica-exposed diversity outbred (DO) mice [59].

Moreover, the inflammatory response after crystalline silica exposure in the lungs of NZBWF1 mice is characterized by extensive perivascular and peri-bronchial lymphoplasmacytic infiltration consisting of IgG-producing plasma cells, and CD45R + B cells and CD3 + T lymphocytes [55, 58, 66]. The spatial architecture of these B and T cell aggregates resembles that of ectopic/tertiary lymphoid tissue/structures (ELT/ELS). Chauhan et al. [65] confirmed these findings in the same mouse strain, but using a single dose of crystalline silica in contrast with multiple instillations in the other studies by the group of Pestka and colleagues. Foster et al. [62] observed ELS in NZB/BINJ, BXSB, MRL and C57BL/6 mice after crystalline silica-exposure. In addition, leukocyte infiltration, granuloma formation, alveolar proteinosis, lymphoid collections, oedema, and scattered haemorrhage were observed as well in crystalline silica-exposed NZB/BINJ, BXSB, MRL and C57BL/6 mice [62].

#### Kidney pathology

Crystalline silica was shown to induced extensive IgG and complement C3 immune complex deposition within glomeruli of NZM 2410 mice [47, 50]. Glomerulonephritis was also reported in crystalline silica-exposed DO mice [59]. These changes can eventually result in kidney nephritis and injury with significant proteinuria in both NZM 2410 and NZBWF1 mice [50, 66, 69].

#### Lymph nodes and spleen

In the spleen and the hepatic lymph node of C57BL/6 mice, an increased percentage of CD4 + Foxp3 + T cells was observed after crystalline silica exposure. Moreover, percentage of CD4 + IL-17A + T cells in spleen significantly increased after crystalline silica exposure in healthy C57BL/6 mice [54].

Ezendam and colleagues [48] found an increase in popliteal lymph node cell numbers after silica exposure in Balb/c mice. In addition, silica increased IgM antibody-secreting B cells, but not IgG1 antibody-secreting B cells in popliteal lymph nodes of exposed mice. However, silica was not able to induced significant germinal centres. Silica induced a significant increase of the absolute number of Th and Tc cells in popliteal lymph nodes.

B1a B and CD4 + T cell numbers were both significantly increased within superficial cervical lymph nodes of crystalline silica-exposed NZM 2410 mice compared with non-exposed control mice [49]. In contrast, the numbers of CD4 + CD25 + T were not influenced by crystalline silica exposure.

#### BAL fluid cytology, (auto)-antibodies and inflammatory biomarkers

Brown et al. [49] observed a significant increase in TNF-α levels in BALF from crystalline silica-exposed NZM 2410 mice. Also in NZBWF1 mice, crystalline silica induced an elevation of TNF-α [55]. In addition, monocyte chemoattractant protein 1 (MCP-1) and IL-6 were elevated as well. Chauhan et al. [65] observed an elevated secretion of IL-1α, IL-1β, IL-18, TNF-α, IL-6, MCP-1, and B cell activation factor (BAFF) in NZBWF1 mice after crystalline silica exposure.

Instillation of crystalline silica in Sprague–Dawley rats resulted in sustained inflammation, indicated by increased in levels of total protein and neutrophils, lactate dehydrogenase activity and β-glucuronidase activity. Besides that, white blood cell counts were elevated in BALF of exposed rats [53].

Crystalline silica was also shown to induce elevation of IgG, IgA, and IgM in crystalline silica exposed NZBWF1 mice [55]. Foster et al. found that anti-DNA IgM and IgG autoantibodies were significantly higher in BALF of silica-exposed C57BL/6, BXSB, and MRL mice compared to non-exposed control mice [62].

Rajasinghe et al. [64] observed robust IgG and IgM autoantibody responses against lupus-associated autoantigens including DNA, histones, ribonucleoprotein, Smith antigen, Ro/SSA, La/SSB, and complement one week after crystalline silica exposure in BALF of NZBWF1 mice. Importantly, crystalline silica also induced autoantibodies to autoantigens associated with rheumatoid arthritis (collagen II, fibrinogen IV, fibrinogen S, fibronectin, and vimentin), Sjögren’s syndrome (α-fodrin), systemic sclerosis (topoisomerase I), vasculitis (MPO and PR3), myositis (Mi-2, TIF1-γ, MDA5), autoimmune hepatitis (LC-1), and celiac disease (TTG).

#### Serum immunoglobulins

Brown and colleagues reported a decrease in overall serum IgG levels [47] and a decrease in serum IgG1 levels [49] of crystalline silica-exposed NZM 2410 mice. Serum IgM levels did not significantly differ from non-exposed control mice [47].

#### Serum autoantibodies

Brown et al. [47] observed elevated levels of serum ANA and anti-histone antibodies in serum of crystalline silica-exposed NZM 2410 mice. The increase in ANA was confirmed by a study by Pfau et al. [21]. In addition, autoantibodies isolated from sera of these mice were found to bind to apoptotic debris derived from alveolar macrophages [21]. Serum ANA and anti-RNP antibodies were also shown to be increased in sodium silicate-exposed Brown Norway rats [51, 52]. Also in subsets of diversity outbred mice, crystalline silica was able to induce serum ANA [59].

Bates et al. [55] observed that crystalline silica can also induce anti-dsDNA and ANA in NZBWF1 mice. Chen et al. [54] observed that also in non-autoimmune prone C57BL6/J mice, crystalline silica exposure can induce both ANA and anti-dsDNA antibodies in serum. Engelmann et al. [57] observed a higher anti-cyclic citrullinated peptides (anti-CCP) antibody titre in F1 crosses of female DBA/1 J and male B10.q mice.

Besides in BALF, Rajasinghe et al. [64] also observed a similar robust IgG and IgM autoantibody responses against the same autoantigens associated with several systemic autoimmune diseases in serum of crystalline silica exposed NZBWF1 mice.

A study by Gonzalez-Quintial et al. [68] observed that a single dose of crystalline silica in C57BL/6 mice did not induce an autoantibody response, while in combination with an infection with the model murine pathogen lymphocytic choriomeningitis virus (LCMV) autoantibodies to several nuclear self-antigens including chromatin, RNP and Sm in serum could be observed.

#### Systemic inflammatory biomarkers

Serum IgG was shown to be decreased in crystalline silica-exposed NZM 2410 mice [47, 49]. Serum IL-17A levels were higher in crystalline silica-exposed C57BL/6 mice compared to non-exposed control mice. In addition, also serum MCP-1, TNF-α, and IL-6 were elevated in crystalline silica-exposed NZBWF1 mice [55].

#### Mechanistic evidence

Besides observations of crystalline silica-induced changes in several parameters related to systemic autoimmunity, some of the included studies provided some more mechanistic insights.

Lescoat et al. [63] reported a decrease in the efferocytosis index of alveolar macrophages from crystalline silica exposed C57BL/6 mice. Efferocytosis is the clearance of apoptotic cells, which is an essential process in the resolution of inflammation. In addition, rottlerin, a protein kinase Cδ (PKCδ)-selective inhibitor that blocks apoptosis, was observed to decrease crystalline silica induced autoimmunity, which gives another indication of the importance of apoptosis in systemic autoimmunity development.

Engelmann et al. [57] examined the abundance of peptidyl arginine deaminase enzymes 2/4 (PAD 2/4) in the lungs of crystalline silica-exposed F1 crosses of female DBA/1 J and male B10.q mice and did not observe higher levels of PAD enzymes compared to non-exposed control mice. PAD 2/4 enzymes are essential for the post-translational modification that replaces an arginine with a citrulline, called citrullination.

Few of the included studies also examined transcriptional changes and gene expression in different tissues, mainly lung and kidney, of crystalline silica exposed mice [60, 61]. Modest transcriptional changes associated with innate and adaptive immune response after a single instillation and higher transcriptional changes after repeated exposure with multiple instillations were observed in crystalline silica-exposed NZBWF1 mice. The time period between 5 and 9-weeks post-instillation seemed to reflect an important transition period where considerable immune gene upregulation in the lung was observed. Importantly, mRNA signatures in lungs of crystalline silica-treated mice over 13 weeks reflected progressive amplification of interferon (IFN)- and chemokine-related gene pathways [61]. Besides in lung, also in kidney tissue, genes associated with inflammation, innate/adaptive immunity, IFN, chemokines, and antigen processing, were alternatively expressed compared to non-exposed control mice.

## Discussion

To gain a better sense of the status of research into silicate-induced autoimmunity, we evaluated reported systemic autoimmune phenotypes, animal models, exposure protocols, and overall quality of the studies, within the framework of a systematic review for animal studies [29] and a ToxR quality assessment [33]. To our knowledge, this is the first evidence-based analysis in this field. Our review showed that both amphibole asbestos and crystalline silica can induce different types of antibodies, both locally and systemically, and that both silicates can induce clinical effects of systemic autoimmunity, specifically of SLE, represented by kidney malfunction. Evidence is more convincing for crystalline silica compared to amphibole asbestos. Chrysotile asbestos, however, was not able to induce ANA or kidney malfunction.

### Suitability and limitations of the experimental models and exposure routes

For this systemic review, we chose to focus on studies using rodents, because the relationship to human biology has been extensively analyzed and toxicology assessment methodology is highly adapted to rodent studies. The current body of literature is characterized by non-uniform exposure and outcome measurements in studies both across and within species.

#### Choice of choice of rodent strain

Although most of the included studies use healthy non-autoimmune prone strains, these studies can be hindered by the complexity of disease susceptibility loci that may or may not be present [70]. To overcome this limitation, researchers have opted to use autoimmune-prone strains, thereby mimicking a genetically predisposed population. In this case, the interest is in the potential of exposure to exacerbate or accelerate disease. One study [59] opted for the use of outbred strains to broaden genetic heterogeneity, providing a better model to study population-based disease features [71–73]. In this study [59], the DO mouse strain was used, which is a heterogeneous stock derived from eight founder strains (A/J, C57BL/6 J, 129S1/SvImJ, NOD/ ShiLtJ, NZO/HiLtJ, CAST/EiJ, PWK/PhJ, and WSB/EiJ), maintained by randomized breeding. These mice exhibit significant phenotypic variability as they capture the same set of allelic variants as the eight founder strains. Therefore, the DO mice model is well suited to capture the wide range of immunological responses as a result of exposure to environmental agents known to induce disease in humans [74, 75].

#### Exposure method

Some of the included studies described a validation for the exposure route to examine the most uniform presence of silica in the lung and the reproducibility, where oropharyngeal/trans-oral instillation seemed to be a better option compared to intratracheal instillation [59], possibly because intratracheal instillation is more likely to affect both the size and quantity of particles reaching the lower respiratory tract by bypassing the pharynx and upper trachea [45, 76, 77]. None of the included studies used an inhalation exposure in which the animals are exposed to an aerosol containing particles, hereby mimicking chronic inhalation exposure in humans. However, because this concerns a whole-body exposure, there is not only inhalation exposure, but also dermal, and subsequently oral exposure. Although inhalation exposure mimics the human situation, the additional oral and dermal exposure makes it difficult to determine which exposure source drives the resulting disease.

#### Choice of particles/fibers

In the asbestos studies, the preference went to amphibole asbestos types, and more specifically to the Libby amphiboles (e.g. Libby 6-Mix). As chrysotile asbestos is the most commonly used form of asbestos, more research might be needed using these fibers.

In contrast with crystalline silica, fabricated stone has yet to be tested for its ability to induce systemic autoimmunity, as this type of exposure is a worldwide problem in humans. The silica content of artificial stone is around 90%, which is much higher compared to natural stone (e.g. average of 30% in granite). Few studies have identified cased on artificial stone-induced silicosis [78, 79]. In addition, Shtraichman et al. found a sevenfold excess in autoimmune features in a database of artificial stone-induced silicosis patients [80]. However, the abilities of artificial stone to induce systemic autoimmunity has not yet been examined in experimental models.

### Mechanistic evidence generated by these studies and limitations

A specific lupus-like-autoimmune reaction could be seen in C57BL/6 mice exposed to amphibole asbestos, resulting in ANA, glomerular damage, and immune complex deposition [37]. These experimental data are in line with the limited epidemiological evidence linking asbestos exposure with autoimmune diseases. A nested case–control study of self-reported SLE and SSc patients from a medically screened general population cohort in Libby, Montana, U.S., showed associations for both diseases with amphibole asbestos exposure [81]. Also hallmarks of lung pathology were observed, such as lung collagen and MCAA, which are associated with excess production of pleural collagen in amphibole asbestos-exposed mice [44]. This is consistent with findings from a cohort study where MCAA were observed in sera of Libby amphibole-exposed subjects [82].

Chrysotile asbestos-exposed animals did not show any increase in autoantibodies or any increase in cytokines other than IL-6.

Data from the one included study using erionite fibers [43] suggests that erionite may behave similarly to amphibole asbestos and may potentially promote the production of autoantibodies commonly seen in people with systemic autoimmune diseases, like ANA. This data suggests that immune effects are fiber-specific, which may influence disease manifestations and severity, with more pronounced autoimmune-inducing effects resulting from amphibole asbestos and erionite fiber exposure, compared to chrysotile asbestos exposure.

Similar to asbestos, a specific lupus-like-autoimmune reaction was observed after (crystalline) silica exposure in several of the included studies, resulting in serum ANA, glomerular damage, and kidney immune complex deposition in different autoimmune-prone mouse strains, such as NZM 2410 and NZBWF1 mice. In healthy mice strains, such as C57BL/6, in contrast, evidence for silica-induced systemic autoimmune effects was less convincing. Kidney lesions and modest leukocyte infiltrations in BALF was observed [55]. In addition, crystalline silica resulted in impaired efferocytosis of alveolar macrophages [63] in C57BL/6 mice. Efferocytosis is a key process in the resolution of inflammation and a defect in this process is reported in macrophages from patients with fibrotic- or autoimmune diseases [83]. When efferocytosis fails, apoptotic cells can rupture, releasing cellular materials that might act as damage-associated molecular patterns (DAMPs) stimulating an autoimmune response. Determining the link between silica exposure and efferocytosis may elucidate the relationship between innate immunity and fibrosis, and eventually, systemic autoimmunity.

In addition to the effects observed in lupus-prone strains, exposure to crystalline silica was able to induce or accelerate systemic autoimmunity in a fraction of DO mice [59]. Based on these findings, exposure of the diversity outbred mouse to silica seems to provide a model of the diverse systemic autoimmune responses of the human population to crystalline silica exposure. As a result, the DO mouse possibly provides a unique opportunity to investigate genetic pathways and pathophysiologic mechanisms critical for promoting silica-associated autoimmune disease and therefore supports the use of this model in investigating population-wide environmental effects.

Although it is hard to compare the different included studies, both amphibole asbestos and crystalline silica can induce (sub-)clinical systemic autoimmune features in different mouse and rat strains. This raises questions regarding a possible common pathway in the induction of systemic autoimmunity after exposure to different types of silicates. However, the ability to draw conclusions from these in vivo rodent studies regarding a common pathway is hindered for multiple reasons. One stumbling block is that the current body of animal-based evidence consists of mainly observational results from studies focusing on the establishment of environmentally-induced models of systemic autoimmune diseases. In addition, the included studies examine a wide variety of variables that are not always examined in both silica- or asbestos-exposed animals.

### Sex effects of silicate-induced autoimmunity

Since most autoimmune diseases occur more often in women than in men [1], it might be interesting to take into account this variable when studying the pathogenesis of autoimmunity in rodents. Unfortunately, research articles investigating the role of crystalline silica and asbestos in the onset of systemic autoimmune diseases actually taking sex effects into account, are limited..

Mayeux et al. [59] reported that major sex differences are reflected in greater lung inflammation, BALF cells and IL-6, and silica induced anti-ENA5 autoantibodies in male DO mice exposed to crystalline silica [27]. Tassinari et al. found that female rats showed a higher reactivity of T cell responses and male rats were found to be more prone to blood cell count reduction. And they confirmed a sex related female sensitivity of Synthetic Amorphous Silicon Dioxide  when it targets the thryoid [39].

For asbestos, Christofidou-Solomidou et al. [46] concluded that in general, immune responses to amphibole asbestos followed similar patterns for male and female mice. In this study male and female mice were used for separate experiments, so differences in the absolute values were observed. They concluded that the data does suggest that future studies should be designed with potential gender differences in mind [34].

### Limitations and strengths of the study

Although the methods of this systematic review followed the Systematic Review Protocol for Animal Intervention Studies, the protocol was not published previous to the performance of the review, which is a limit of the study. However, all the screening steps, the ToxR tool assessment and the data extraction were performed by two reviewers (independently for all steps except for the ToxR tool), which is a strength of the current study.

### Perspectives for the assessment of autoimmunity in environmental toxicology

More knowledge is needed on the role of silicates in the development of autoimmune diseases to propose complete and specific pathways of environmentally-induced systemic autoimmunity. Such information will have a significant impact on the development of preventive measures and therapeutic agents. Although the data gained from experimental animals has yielded important insights, other experimental designs and approaches will be needed to fill the remaining gaps in the literature. The synergistic effects of multiple exposure and genetic predisposition are two areas that are crucial to a complete understanding of autoimmune disease pathogenesis and more precisely to determine individual risk of developing disease.

The multi-hit model of autoimmunity, where exposure to different environmental factors acting on distinct immunostimulatory pathways complements limited genetic predisposition, can increase the risk of autoimmunity above a critical threshold. One of the included studies [68] examined the synergistic effects of chronic virus infection to the LMCV virus and crystalline silica exposure in C57BL/6 mice. As discussed previously, crystalline silica alone is not capable of inducing systemic autoimmunity in these mice, and neither is the LCMV virus [84]. The results of this study raises the question of whether several exposure compounds and genetic factors synergize by activating distinct immunostimulatory pathways, together possibly leading to a more effective break of tolerance, earlier disease onset, and enhanced severity? These findings create awareness of the fact that we should consider the hypothesis that a baseline exposure or a first triggering event, such as a virus infection, in combination with other triggers in later life, such as occupational crystalline silica-exposure, can lead to disease. This is particularly relevant in the current COVID-19 pandemic, where a large part of the population has been infected. Recent studies have shown the presence of subclinical autoimmunity, mainly autoantibodies, in both recovered and severe COVID-19 patients [85, 86]. Whether additional triggers, such as smoking or inorganic dust containing silicates, will result in autoimmune disease is unclear, but worthy of continued study. Secondly, there is a need for a more detailed characterization of the effects of particulate exposure on the immune system, especially the contribution of the inflammasome, inflammatory cytokines, toll-like receptors (TLR), and modification of self-antigens by post-translational modifications such as citrullination.

The findings from this analysis of studies on silicate exposure and systemic autoimmunity highlight the need for additional well-designed animal studies that examine the effects of environmental stressors alone or in combination in genetically well-defined species/strains to pick up genetic and sex dependent susceptibility, and the included endpoints should allow a mechanistic focus to unravel the molecular and cellular events required for the development of autoimmunity following exposure.

## Supplementary Information

**Additional file 1**: Selection criteria. Inclusion and exclusion criteria

**Additional file 2: Table 1**: Search strings. Search strings for PubMed and Embase

**Additional file 3: Table 2**: ToxR tool questions. The 21 questions used for the ToxR quality assessment

**Additional file 4: Table 3**: Data extraction table. Data extraction table with data on animal models, exposure protocols and key endpoints from the included studies.

**Additional file 5: Table 4**: ToxR Tool results. Results of the ToxR quality assessment for the included studies.

## Data Availability

Not applicable.

## Abbreviations

ADs: Autoimmune diseases
T1D: Type 1 Diabetes
SLE: Systemic lupus erythematosus
RA: Rheumatoid arthritis
SSc: Systemic sclerosis
SAIDs: Systemic autoimmune diseases
IARC: International Agency for Research on Cancer
ANA: Antinuclear antibodies
ANCA: Antineutrophil cytoplasmic antibody
PECO: Population, exposure, comparator, outcome
MeSH: Medical Subject Headings
NIEHS: National Institutes for Environmental health Sciences
ToxRTool: Tociological data Reliability Assessment Tool
NZM: New Zealand Mixed
LDH: Lactate dehydrogenase
BALF: Bronchoalveolar lavage fluid anti-CCP anti-cyclic citrullinated peptides
PG-PS: Peptidoglycan-polysaccharide
SMA: Smooth muscle alpha-actin
MCAA: Mesothelial cell autoantibodies
IgG: Immunoglobulin G
IgM: Immunoglobulin M
Th17: T helper 17
IL-17: Interleukin-17
IL-6: Interleukin-6
TNF-α: Tumor necrosis factor alpha
DO: Diversity outbred
ELT/ELS: Ectopic lymphoid tissue/structures
MCP-1: Monocyte chemoattractant protein 1
BAFF: B cell activation factor
LCMV: Lymphocytic choriomeningitis virus
PKCδ: Protein kinase C delta
PAD2/4: Peptidyl arginine deaminase enzymes 2/4
IFN: Interferon
DAMPs: Damage-associated molecular patterns
TLR: Toll-like receptors
