# Supplementary material for: Exposure to silicates and systemic autoimmune-related outcomes in rodents: a systematic review
Source: Part Fibre Toxicol. 2022 Jan 7;19:4. doi: 10.1186/s12989-021-00439-6 (PMC8739508; doi:10.1186/s12989-021-00439-6)
Supplement: Supplementary file 1 — Additional file 1: Selection criteria. Inclusion and exclusion criteria [file 12989_2021_439_MOESM1_ESM.docx]

Selection criteria

# Title/abstract selection:

Inclusion:

1. *In vivo* animal intervention study using **rodents**
   1. The intervention has to take place in the animals (e.g. dosing with particles)
   2. In vitro experiments are okay if dosing with silicates took place in the animals.
2. Study assesses **effects of exposure** (can be all different types of endpoints, adverse and positive) to (one or more of) the selected occupational xenobiotic compounds (silica/asbestos)
   1. Also include if silica is used as a control situation for a therapy, as essential data may come from these studies.
   2. Silica can occur in different forms. Based on literature search, following forms are included:
      1. Crystalline silica forms: α/β-quartz, tridimyte, cristobalite, keatite, moganite, coesite, stishovite, seifertite, melanophlogite, fibrous W-silica, 2D silica
      2. Amorphous silica forms: opal, quartz glass, tachylite, obsidian, tektite, lechatelierite, geyserite
   3. Different types of asbestos: serpentine (chrysotile), amphibole (amosite, crocidolite, tremolite)
      1. Also papers assessing effects of talc are included at this points, as they might be contaminated with tremolite.

Exclusion:

1. If it has no abstract available
2. If it is a review paper
3. Other animals than rodents were used
4. Intervention was performed on rodent cells in vitro
5. Silica can occur in different forms. Based on literature search, following forms are excluded:
   1. Clay
   2. Kaolin or other food supplements that contain silica

# Full text screening:

Inclusion:

1. *In vivo* animal intervention study using **rodents**
   1. The intervention has to take place in the animals (e.g. dosing with particles)
   2. In vitro experiments are okay if dosing with silicates took place in the animals.
2. Study assesses **effects of exposure** to (one or more of) the selected occupational xenobiotic compounds (silica/asbestos)
   1. Also include if silica is used as a control situation for a therapy, as essential data may come from these studies.
   2. Silica can occur in different forms. Based on literature search, following forms are included:
      1. Crystalline silica forms: α/β-quartz, tridimyte, cristobalite, keatite, moganite, coesite, stishovite, seifertite, melanophlogite, fibrous W-silica, 2D silica
      2. Amorphous silica forms: opal, quartz glass, tachylite, obsidian, tektite, lechatelierite, geyserite
   3. Different types of asbestos: serpentine (chrysotile), amphibole (amosite, crocidolite, tremolite)
      1. Also papers assessing effects of talc are included at this points, as they might be contaminated with tremolite.
3. Studies that assess **systemic autoimmune outcomes** (*Systemic autoimmune diseases*: systemic sclerosis, systemic lupus erythematosus, rheumatoid arthritis, Sjögren’s syndrome, antineutrophil cytoplasmic antibody (ANCA)-associated vasculitis, autoimmune myositis / *Systemic autoimmunity features*: kidney pathology/glomerulonephritis, lung pathology, autoantibodies, changes in serum immunoglobulins, changes in serum cytokines, proteinuria, skin involvement, joint involvement)

OR

Studies that investigate **mechanisms of autoimmunity in general/systemic autoimmunity** (not studies focused on mechanisms of organ-specific autoimmunity)

1. Publication type (only journal articles that present original unique data, so no reviews etc.)

Exclusion:

1. If it has no abstract available
2. If it is a review paper
3. Other animals than rodents were used
4. Intervention was performed on rodent cells in vitro
5. Silica can occur in different forms. Based on literature search, following forms are excluded:
   1. Clay
   2. Kaolin or other food supplements that contain silica
