# Supplementary material for: Exposure to silicates and systemic autoimmune-related outcomes in rodents: a systematic review
Source: Part Fibre Toxicol. 2022 Jan 7;19:4. doi: 10.1186/s12989-021-00439-6 (PMC8739508; doi:10.1186/s12989-021-00439-6)
Supplement: Supplementary file 2 — Additional file 2: Table 1: Search strings. Search strings for PubMed and Embase [file 12989_2021_439_MOESM2_ESM.docx]

**Search string Pubmed**

| ("Rodentia"[Mesh] OR "Models, Animal"[Mesh:NoExp] OR “Arthritis, Experimental”[Mesh] OR “Diabetes Mellitus, Experimental”[Mesh] OR "Animal Experiment*"[tiab] OR "Animal models"[tiab] OR rodent*[tiab] OR rat[tiab] OR rats[tiab] OR rattus[tiab] OR mice[tiab] OR mouse[tiab] OR murine[tiab] OR mus[tiab] OR "guinea pig"[tiab] OR "guinea pigs"[tiab] OR cavia*[tiab] OR hamster*[tiab] OR muridae[tiab] OR cricetinae[tiab]) | ("Rodentia"[Mesh] OR "Models, Animal"[Mesh:NoExp] OR “Arthritis, Experimental”[Mesh] OR "Animal Experiment*"[tiab] OR "Animal model*"[tiab] OR rodent*[tiab] OR rat[tiab] OR rats[tiab] OR rattus[tiab] OR mice[tiab] OR mouse[tiab] OR murine[tiab] OR mus[tiab] OR "guinea pig"[tiab] OR "guinea pigs"[tiab] OR cavia*[tiab] OR hamster*[tiab] OR muridae[tiab] OR cricetinae[tiab] OR "New Zealand Mixed"[tiab] OR BXSB[tiab] OR "NZB W F1"[tiab] OR MRL-Fas*[tiab] OR "DBA 1"[tiab] OR SNF1[tiab] OR "NFS sld"[tiab] OR Sprague-Dawley[tiab] OR "Lewis rat*"[tiab] OR "Dark august"[tiab] OR NZM2410[tiab] OR NZBWF1[tiab] OR "bleomycin-mouse model"[tiab] OR "bleomycin-induced"[tiab] OR "HOCl mouse model"[tiab] OR "HOCl-induced"[tiab] OR "Hypochlorous acid"[tiab] OR Fra-2[tiab] OR "collagen-induced"[tiab])) |
| --- | --- |
| ("Silicates"[Mesh] OR "Silicon Dioxide"[Mesh] OR "metals, heavy"[Mesh] OR "Pesticides"[Mesh] OR "Solvents"[Mesh] OR "Hair dyes"[Mesh] OR (Silica[tiab] OR Silicate*[tiab] OR "Silicon dioxide"[tiab] OR Quartz[tiab] OR "Heavy metal*"[tiab] OR Pesticide*[tiab] OR Asbestos[tiab] OR "Organic dust"[tiab] OR Solvent*[tiab] OR "Hair dyes"[tiab] OR Cadmium[tiab] OR Mercury[tiab] OR Herbicides[tiab] OR Insecticides[tiab] OR Amphibole[tiab] OR Chrysotile[tiab]) OR Crysotile[tiab]) | ("Silicates"[Mesh] OR "Silicon Dioxide"[Mesh] OR Silica[tiab] OR Silicate*[tiab] OR "Silicon dioxide"[tiab] OR Quartz[tiab] OR DQ12[tiab] OR "min u sil"[tiab] OR Asbestos[tiab] OR Amphibole[tiab] OR Chrysotile[tiab] OR Crysotile[tiab] OR Crocidolite[tiab] OR Serpentine[tiab] OR Amosite[tiab] OR Tremolite[tiab] OR "mineral dust"[tiab]) |
| ("autoimmunity"[Mesh] OR "autoantibodies"[Mesh] OR “autoimmune diseases”[Mesh] OR "autoimmun*"[tiab] OR "auto immun*"[tiab] OR "autoantibod*"[tiab] OR "auto antibod*"[tiab] OR "antinuclear antibod*"[tiab] OR "anti nuclear antibod*"[tiab] OR "anti-DNA antibod*"[tiab] OR "rheumatoid arthritis"[tiab] OR "lupus"[tiab] OR "SLE"[tiab] OR "systemic sclerosis"[tiab] OR "scleroderma"[tiab] OR "autoantigen*"[tiab] OR "auto antigen*"[tiab] OR "diabetes type 1"[tiab] OR "type 1 diabetes"[tiab] OR "myasthenia gravis"[tiab] OR "Sjogrens syndrome"[tiab] OR "autoimmune hepatitis"[tiab] OR "juvenile arthritis"[tiab] OR "Glomerulonephritis"[tiab] OR "Graves disease"[tiab]) | ("autoimmunity"[Mesh] OR "autoantibodies"[Mesh] OR “autoimmune diseases”[Mesh] OR "autoimmun*"[tiab] OR "auto immun*"[tiab] OR "autoantibod*"[tiab] OR "auto antibod*"[tiab] OR "antinuclear antibod*"[tiab] OR "anti nuclear antibod*"[tiab] OR "anti-DNA antibod*"[tiab] OR "rheumatoid arthritis"[tiab] OR "lupus"[tiab] OR "SLE"[tiab] OR "systemic sclerosis"[tiab] OR "scleroderma"[tiab] OR "autoantigen*"[tiab] OR "auto antigen*"[tiab] OR "Sjogren's Syndrome"[Mesh] OR "Sjogren s syndrome"[tiab] OR "Sjogrens syndrome"[tiab] OR "Glomerulonephritis"[tiab] OR “Anti-neutrophil cytoplasmic antibody-associated vasculitis”[tiab] OR "ANCA-vasculitis"[tiab] OR "ANCA associated vasculitis"[tiab] OR myositis[tiab] OR “Mixed Connective Tissue Disease”[Mesh] OR “Mixed Connective Tissue Disease”[tiab] OR "New Zealand Mixed"[tiab] OR BXSB[tiab] OR "NZB W F1"[tiab] OR MRL-Fas*[tiab] OR "DBA 1"[tiab] OR SNF1[tiab] OR "NFS sld"[tiab] OR Sprague-Dawley[tiab] OR "Lewis rat*"[tiab] OR "Dark august"[tiab] OR NZM2410[tiab] OR NZBWF1[tiab] OR "bleomycin-mouse model"[tiab] OR "bleomycin-induced"[tiab] OR "HOCl mouse model"[tiab] OR "HOCl-induced"[tiab] OR "Hypochlorous acid"[tiab] OR Fra-2[tiab] OR "collagen-induced"[tiab]) |
| (english[la]) NOT ("Review"[Publication Type]) | (english[la]) NOT ("Review"[Publication Type]) |

*Result: N = 428 (17-09-2021)*

**Search string Embase**

| ('rodent'/exp OR 'rodent model'/exp OR 'rodent*':ti,ab,kw OR 'animal model':ti,ab,kw OR 'animal experiment*':ti,ab,kw OR 'mice':ti,ab,kw OR 'mouse':ti,ab,kw OR 'mus':ti,ab,kw OR 'rat':ti,ab,kw OR 'rats':ti,ab,kw OR 'rattus':ti,ab,kw OR 'murine':ti,ab,kw OR 'hamster*':ti,ab,kw OR 'guinea pig':ti,ab,kw OR 'guinea pigs':ti,ab,kw OR 'cavia*':ti,ab,kw OR 'muridae':ti,ab,kw OR 'cricetinae':ti,ab,kw) | ('rodent'/exp OR 'rodent model'/exp OR 'rodent*':ti,ab,kw OR 'animal model':ti,ab,kw OR 'animal experiment*':ti,ab,kw OR 'mice':ti,ab,kw OR 'mouse':ti,ab,kw OR 'mus':ti,ab,kw OR 'rat':ti,ab,kw OR 'rats':ti,ab,kw OR 'rattus':ti,ab,kw OR 'murine':ti,ab,kw OR 'hamster*':ti,ab,kw OR 'guinea pig':ti,ab,kw OR 'guinea pigs':ti,ab,kw OR 'cavia*':ti,ab,kw OR 'muridae':ti,ab,kw OR 'cricetinae':ti,ab,kw OR 'New Zealand Mixed':ti,ab,kw OR BXSB:ti,ab,kw OR 'NZB W F1':ti,ab,kw OR 'MRL-Fas*':ti,ab,kw OR 'DBA 1':ti,ab,kw OR SNF1:ti,ab,kw OR 'NFS sld':ti,ab,kw OR Sprague-Dawley:ti,ab,kw OR 'Lewis rat*':ti,ab,kw OR 'Dark august':ti,ab,kw OR NZM2410:ti,ab,kw OR NZBFW1:ti,ab,kw OR 'bleomycin-mouse model':ti,ab,kw OR 'bleomycin-induced':ti,ab,kw OR 'HOCl mouse model':ti,ab,kw OR 'HOCl-induced':ti,ab,kw OR 'hypochlorous acid':ti,ab,kw OR Fra-2:ti,ab,kw OR collagen-induced:ti,ab,kw) |
| --- | --- |
| ('silicon dioxide'/exp OR 'asbestos'/exp OR 'asbestos fiber'/exp OR 'amphibole'/exp OR 'amosite'/exp OR 'chrysotile'/exp OR 'heavy metal'/exp OR 'pesticide'/exp OR 'solvent'/exp OR 'hair dye'/exp OR 'silica':ti,ab,kw OR 'quartz':ti,ab,kw OR 'silicon dioxide':ti,ab,kw OR 'asbestos':ti,ab,kw OR 'amphibole':ti,ab,kw OR 'amosite':ti,ab,kw OR 'chrysotile':ti,ab,kw OR 'crysotile':ti,ab,kw OR 'heavy metal*':ti,ab,kw OR 'cadmium':ti,ab,kw OR 'mercury':ti,ab,kw OR 'pesticide*':ti,ab,kw OR 'solvent*':ti,ab,kw OR 'hair dye*':ti,ab,kw) | ('silicon dioxide'/exp OR 'asbestos'/exp OR 'asbestos fiber'/exp OR 'amphibole'/exp OR 'amosite'/exp OR 'chrysotile'/exp OR 'silica':ti,ab,kw OR 'quartz':ti,ab,kw OR 'silicon dioxide':ti,ab,kw OR 'asbestos':ti,ab,kw OR 'amphibole':ti,ab,kw OR 'amosite':ti,ab,kw OR 'chrysotile':ti,ab,kw OR 'crysotile':ti,ab,kw OR 'silicates":ti,ab,kw OR 'DQ12':ti,ab,kw OR 'tremolite':ti,ab,kw OR 'crocidolite':ti,ab,kw OR 'serpentine':ti,ab,kw OR 'mineral dust':ti,ab,kw) |
| ('autoantibody'/exp OR 'autoimmunity'/exp OR 'autoimmune disease'/exp OR 'autoantibod*':ti,ab,kw OR 'autoantigen':ti,ab,kw OR 'auto antigen':ti,ab,kw OR 'anti-dna antibod*':ti,ab,kw OR 'antinuclear antibod*':ti,ab,kw OR 'auto antibod*':ti,ab,kw OR 'rheumatoid arthritis':ti,ab,kw OR 'lupus':ti,ab,kw OR 'sle':ti,ab,kw OR 'systemic sclerosis':ti,ab,kw OR 'scleroderma':ti,ab,kw OR 'diabetes type 1':ti,ab,kw OR 'type 1 diabetes':ti,ab,kw OR 'myasthenia gravis':ti,ab,kw OR 'Sjogrens syndrome':ti,ab,kw OR 'autoimmune hepatitis':ti,ab,kw OR 'juvenile arthritis':ti,ab,kw OR 'glomerulonephritis':ti,ab,kw OR 'graves disease':ti,ab,kw) | ('autoantibody'/exp OR 'autoimmunity'/exp OR 'autoimmune disease'/exp OR 'myositis'/exp OR 'autoantibod*':ti,ab,kw OR 'autoimmunity':ti,ab,kw OR 'autoantigen':ti,ab,kw OR 'auto antigen':ti,ab,kw OR 'anti-dna antibod*':ti,ab,kw OR 'antinuclear antibod*':ti,ab,kw OR 'anti-nuclear antibod*':ti,ab,kw OR 'auto antibod*':ti,ab,kw OR 'rheumatoid arthritis':ti,ab,kw OR 'lupus':ti,ab,kw OR 'SLE':ti,ab,kw OR 'systemic sclerosis':ti,ab,kw OR 'scleroderma':ti,ab,kw OR 'Sjogrens syndrome':ti,ab,kw OR 'Sjögrens syndrome':ti,ab,kw OR 'Sjögren s syndrome':ti,ab,kw OR 'Sjögrens syndrome':ti,ab,kw OR 'anti-neutrophil cytoplasmic antibody-associated vasculitis':ti,ab,kw OR 'ANCA-vasculitis':ti,ab,kw OR 'ANCA-associated vasculitis':ti,ab,kw OR 'autoimmune myositis':ti,ab,kw OR 'inflammatory myositis':ti,ab,kw OR 'New Zealand Mixed':ti,ab,kw OR BXSB:ti,ab,kw OR 'NZB W F1':ti,ab,kw OR 'MRL-Fas*':ti,ab,kw OR 'DBA 1':ti,ab,kw OR SNF1:ti,ab,kw OR 'NFS sld':ti,ab,kw OR Sprague-Dawley:ti,ab,kw OR 'Lewis rat*':ti,ab,kw OR 'Dark august':ti,ab,kw OR NZM2410:ti,ab,kw OR NZBFW1:ti,ab,kw OR 'bleomycin-mouse model':ti,ab,kw OR 'bleomycin-induced':ti,ab,kw OR 'HOCl mouse model':ti,ab,kw OR 'HOCl-induced':ti,ab,kw OR 'hypochlorous acid':ti,ab,kw OR Fra-2:ti,ab,kw OR collagen-induced:ti,ab,kw OR 'glomerulonephritis':ti,ab,kw) |
| NOT ('conference abstract':it OR 'review':it) AND 'english':la | NOT ('conference abstract':it OR 'review':it) AND 'english':la |

*Result: N = 320 (17-09-2021)*
